# Case of Incarcerated Femoral Hernia

**Published:** 1807-07

**Authors:** A. Bellott

**Affiliations:** Oldham, Lancashire


### *Case of Incarcerated Femoral Hernia;* by Mr. A. Bellott, *of Oldham, Lancashire.*

On Sept. 23, 1806, I was requested to visit Lewis Buckley, who was labouring under symptoms of strangulated hernia; he informed me, that on Monday the 14th instant, after violent exercise at Fives, severe pains came on at the tumour, and also about the navel, which were soon succeeded by sickness and vomiting, with a complete obstruction of the bowels; these symptoms had continued ever since. By the advice of some Medical Practitioners, and others, he had taken repeated doses of castor oil, and other purgatives; he had also used the warm bath; different kinds of glysters had likewise been administered, by which he had voided a small quantity of fæces, but without affording any relief. Having continued in this situation for nine days, the case was now considered as hopeless by his friends; he appeared rapidly approaching towards dissolution, and no time was to be lost. The first thing I attempted was, a reduction by the taxis, which was applied as long as was thought prudent, though with little hopes of success; for, previous to any attempt being made, the patient informed me, he had had the rupture ten or twelve years, and for several years past it had been constantly down; though repeated trials were made, he never could get it up entirely. After the attempts to reduce it be ng fruitless, and all medicines rejected by the stomach, he operation was then proposed, as being the dernier resource, and the only means which would give the patient a chance of recovery; this, however, was not immediately immediately consented to by his friends, they believing he would die, and the operation would put him to a great deal of pain, and probably hasten his end. I then ordered some castor oil, with the infusion of tobacco for glysters, and visited again in the afternoon. The vomiting had recurred, and the stomach rejected every thing that was taken; still I could not gain consent to operate. As we could not stand still in this case, some cathartic pills were then ordered, and I requested to be informed how he went on; the next morning his friends came, saying, that he wished the operation to be performed, and desired me to visit him again.

I requested a medical friend to accompany me; when we arrived, the obstacles, which we expected to find entirely removed, were again increased; some of his relations being particularly averse to the operation, obliged us to postpone it. The cathartic pills before ordered (but neglected to be taken) were now given, and we agreed to visit him again in the afternoon; the pills, however, only tended to increase the symptoms, as the obstruction was complete. The vomiting was now so excessive, that a quantity of stercoraceous matter was thrown up, his pulse scarcely to be numerated, running 140 or more, with cold clammy sweats, and hiccup. These symptoms, in such cases, certainly are the forerunners of death, and denote a speedy dissolution. I was determined, if possible, to perform the operation, though the chance of success was now very small. More than an hour was lost at this critical period, before we could gain consent; at length the friends agreed to leave it entirely to the patient, whose consent was readily obtained. He was immediately placed upon a table of convenient height, his head and shoulders a little raised by pillows, the legs overhanging the edge of the table, and the feet supported on each side. The hair being shaved off, an incision was made, about 7 inches long, nearly over the middle of the tumour, but rather more towards the pubis, thro' the skin; the fascia, which next comes in view, and is in general very strong, was here very thin. The fascia and cellular membrane were cautiously divided the whole length of the tumour, which exposed the hernial sac and ligamentum Poupartii, to which the sac was adhering. With some difficulty it was separated, so as to admit the finger, and the ligament dilated, with Pott's bistoury introduced upon the finger, cutting obliquely downwards and towards the os pubis, afterwards cautiously tearing asunder the adhesions between the the sac and ligament all round. I then drew down the tumour a little, which brought to view the mouth of the sac. Here there appeared to be considerable stricture. I several times attempted to raise the sac from its contents, in order by slight scratches to make an opening into it; but this was ineffectual, as a complete adhesion had taken place between the sac and its contents, throughout the whole of the tumour. At length I succeeded in making a small opening a little above the mouth of the sac; I then introduced a bent probe, and broke down the adhesion to the bottom of the tumour. The grooved director was now easily passed the same way, from above downwards; the stricture and the whole length of the sac were divided upon the director. There were now exposed a portion of omentum and also intestine, adhering together through the whole extent of the sac. The omentum and intestine were cautiously separated, and the intestine, which was much inflamed, returned into the abdomen. The omentum had no marks of mortification, but was much indurated. I hesitated whether to cut a portion of it away, or return the whole. We concluded, however, to return it just within the ligament, thinking it would serve as a barrier to the intestine, and in all probability an adhesion might take place, so as to prevent any future protrusion. The wound was then closed by three sutures and the adhesive plaster, and the patient put to bed. In the course of two hours, a large quantity of fæces was dicharged; an opiate was given, and the patient got several hours comfortable sleep.

On the fourth day, the dressings were removed, and the wound looked very well, though the healing proved afterwards rather tedious, from a sinus which discharged a good deal of matter; he wore a bandage with a compress, which contributed much towards healing the wound; he took the bark at the same time, keeping the bowels regular; and the wound was completely healed in the course of five weeks. He now remains perfectly well; wears no truss, nor has there been the slightest appearance of further protrusion.

P. S. The last person called in this case, before the operation was concluded upon, was one of the many pretenders which we have in this country; he had given the patient 45 of his pills, which he deals out pretty liberally, as a sovereign remedy in almost all diseases; finding no amendment he then left him, and pronounced him a dead a dead man, which must inevitably have been the case had the operation not been performed.—Cases of this nature prove the necessity of a medical reform; and call loudly for legislative interference, to prevent ignorant pretenders from imposing upon the public and sacrificing the lives of his Majesty's subjects.

*June* 3, 1807.

